# Imaging findings of a case of peliosis hepatis mimicking malignancies

**DOI:** 10.1016/j.radcr.2024.06.064

**Published:** 2024-07-20

**Authors:** Noemi Pucci, Flavia Chirico, Maria Pitaro, Eliseo Picchi, Valerio Da Ros, Valentina Ferrazzoli, Luca Toti, Silvia Minosse, Francesco Garaci, Francesca Di Giuliano

**Affiliations:** a Diagnostic Imaging Unit, Department of Biomedicine and Prevention, University of Rome Tor Vergata, Viale Oxford 81, Rome 00133, Italy; b Neuroradiology Unit, Department of Biomedicine and Prevention, University of Rome Tor Vergata, Via Montpellier 1, Rome 00133, Italy; c Department of Surgical Sciences, Tor Vergata University, Via Montpellier 1, Rome 00133, Italy; d San Raffaele Cassino, via Gaetano di Biasio 1, Cassino 03043, Italy

**Keywords:** Peliosis hepatis, MRI, CT, Hepatic lesion, Hepatobiliary-specific contrast agent

## Abstract

Peliosis hepatis (PH) is a rare benign pathological entity characterised by dilatation of the hepatic sinusoids. It has been reported to be associated with infection or malignancy, but the aetiology of PH remains unknown. Distinguishing PH from other malignancies can be difficult on imaging studies. This case report describes the incidental finding of PH in a patient undergoing a cardiac computed tomography (CT) scan at our institution.

The CT scan incidentally revealed areas of altered density in the liver on the abdominal scans, requiring detailed liver diagnostic studies for better characterisation.

## Introduction

Peliosis hepatis (PH) is a vascular disorder characterized by the proliferation of hepatic sinusoids leading to congestion of the capillary bed and cavities within the liver. This condition is typically discovered incidentally during autopsy or abdominal examination and often remains untreated. The aetiology of peliosis remains unclear. Various investigators suggest dilatation of the central vein of the hepatic lobules, direct disruption of the sinusoidal borders, obstruction of hepatic outflow at the sinusoidal level, or hepatocellular necrosis leading to cavitation as possible underlying mechanisms. PH can be idiopathic, but it is frequently induced by drugs, infections, autoimmune diseases, transplants, hematological disorders, and cancer (such as hepatocellular carcinoma) [1,2]. Although focal PH has been documented, lesions usually involve the entire liver. The natural history of peliosis hepatis often involves regression after cessation of drug use, steroid therapy, or resolution of an associated infectious disease. However, there have been reports of progression to portal hypertension and liver failure and hemorrhagic complication [[3], [4], [5]].

The identification of PH lesions can be achieved through a variety of imaging modalities, including sonography, CT, magnetic resonance imaging (MRI), and angiography. However, the observed appearances are nonspecific, with variable enhancement patterns, which can make the diagnosis challenging. In the majority of cases, there are multiple lesions, which may be either a few large lesions or several small lesions [3,6].

### Case

A 61-year-old woman underwent a cardiac CT scan in our diagnostic imaging department. Incidental findings of altered liver density were noted on abdominal scans. The patient was asymptomatic, and hematological and biochemical examinations, including indices of cholestasis, cytolysis, pancreatic enzymes, inflammatory markers, and blood cell count, were within normal ranges.

The patient did not have a history of abdominal diseases, surgeries, diabetes, or the use of drugs such as steroids, antibiotics, and hormonal therapies. Additionally, no infections such as human immunodeficiency virus (HIV), hepatitis, hematopoietic system diseases, or tuberculosis were detected.

**Fig. 1:**
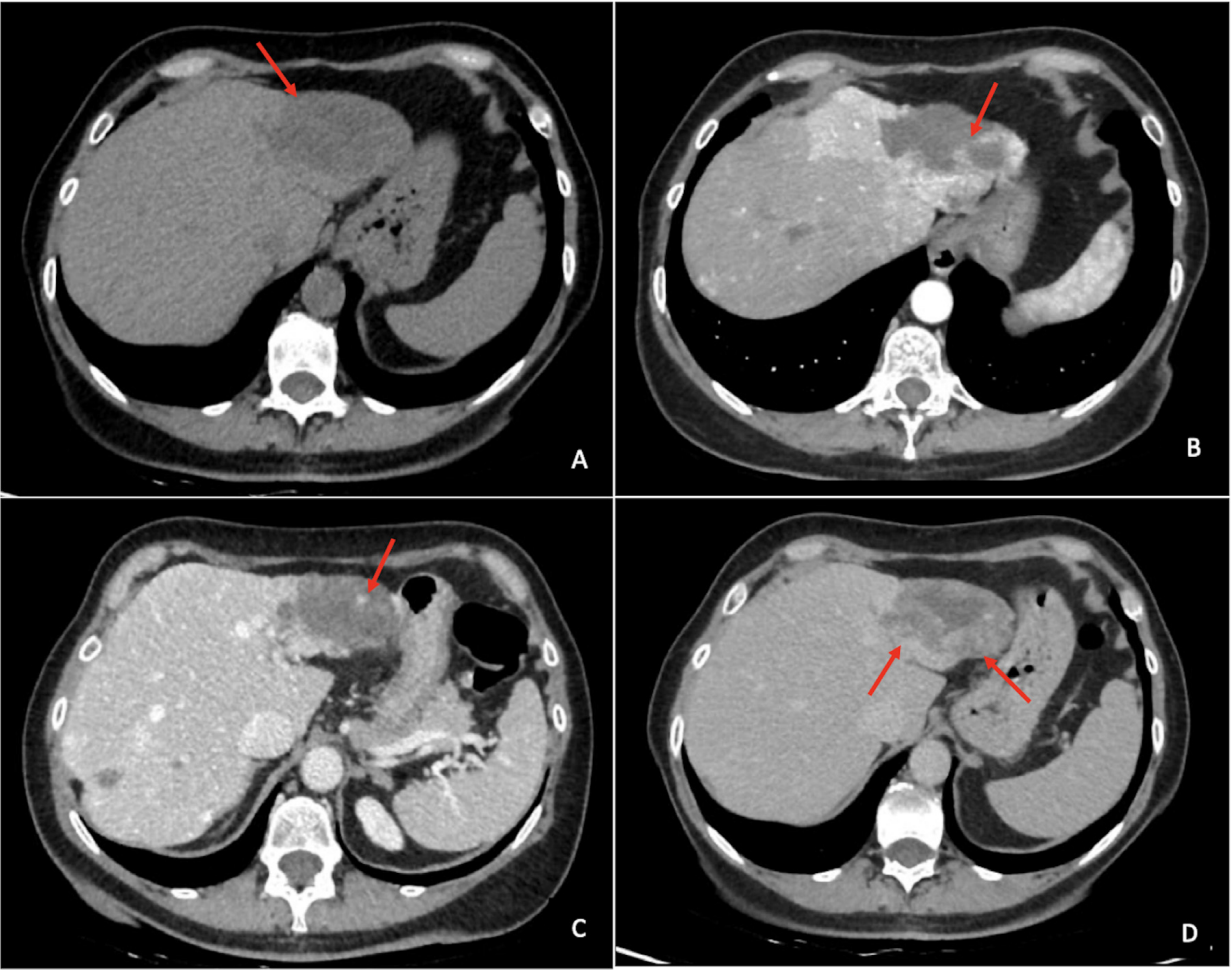
Computed tomography images. An area with polycyclic margins at the II-III hepatic segment is observed, accompanied by evidence of an 8 cm formation in the context of inhomogeneous density in basal conditions (A). This is further characterised by the presence of central vascular pooling evident in the arterial (B) and portal (C) postcontrastographic phases. Subsequently, progressive, irregular and incomplete enhancement of the peripheral portions is observed in the late phase at 7 minutes, with a centrifugal enhancement pattern (D).

**Fig. 2:**
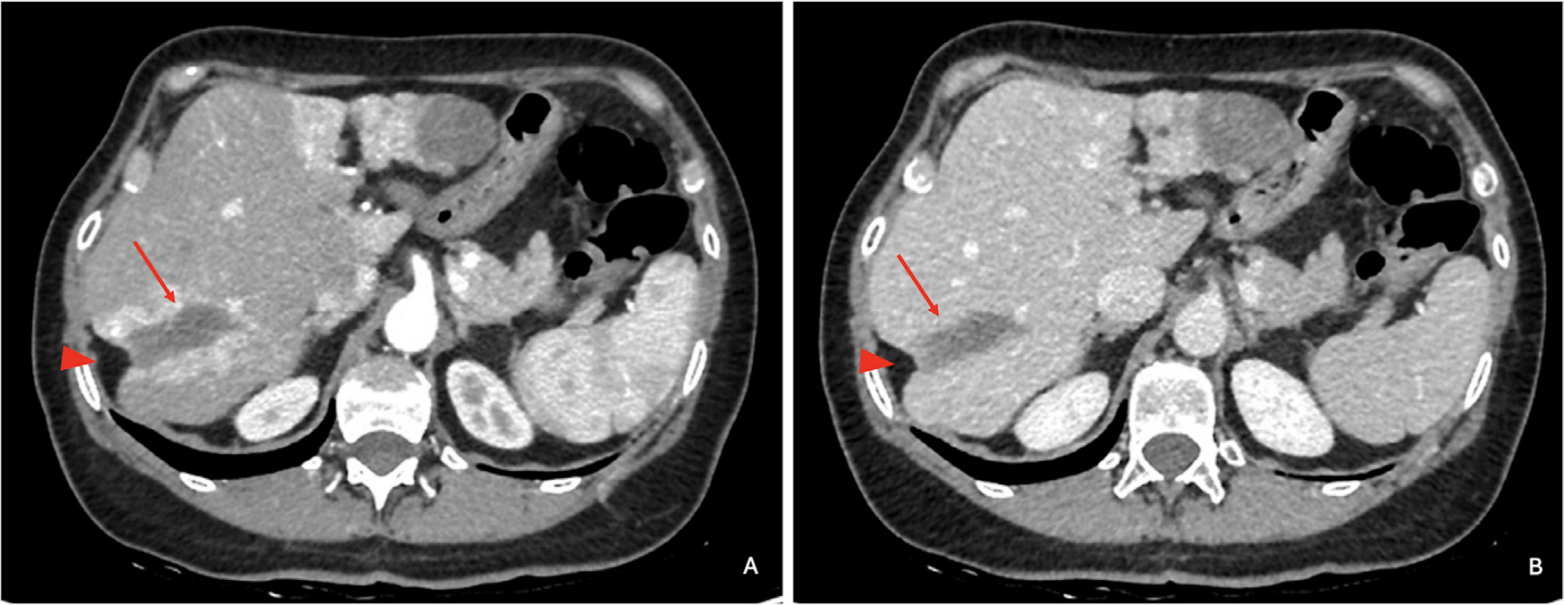
Computed tomography images. An oblong formation at the VII hepatic segment (arrow), measuring 50 × 20 mm, is observed to cause distortion of the capsular profile (arrow head) in both the arterial (A) and venous phases (B).

The CT scan was extended to include abdominal imaging with multiphasic imaging and showed that the liver was within normal dimensions, but there was hypertrophy of the right hemiportion and hypotrophy of the left hemiportion. At the level of the II/III hepatic segment, an 8 cm formation with nonhomogeneous density was observed, showing a central vascular pool in the early contrastographic phase, followed by irregular, progressive and incomplete enhancement of the peripheral portions visible in the late phase acquired at 7 minutes (Fig. 1). The CT scan revealed a further similar 50 × 20 mm “oblong” area of low attenuation in the VII hepatic segment causing distortion of the capsular profile (Fig. 2). Capsular retraction raised diagnostic suspicion, including malignant lesions such as cholangiocarcinoma, and prompted further diagnostic investigation.

**Fig. 3:**
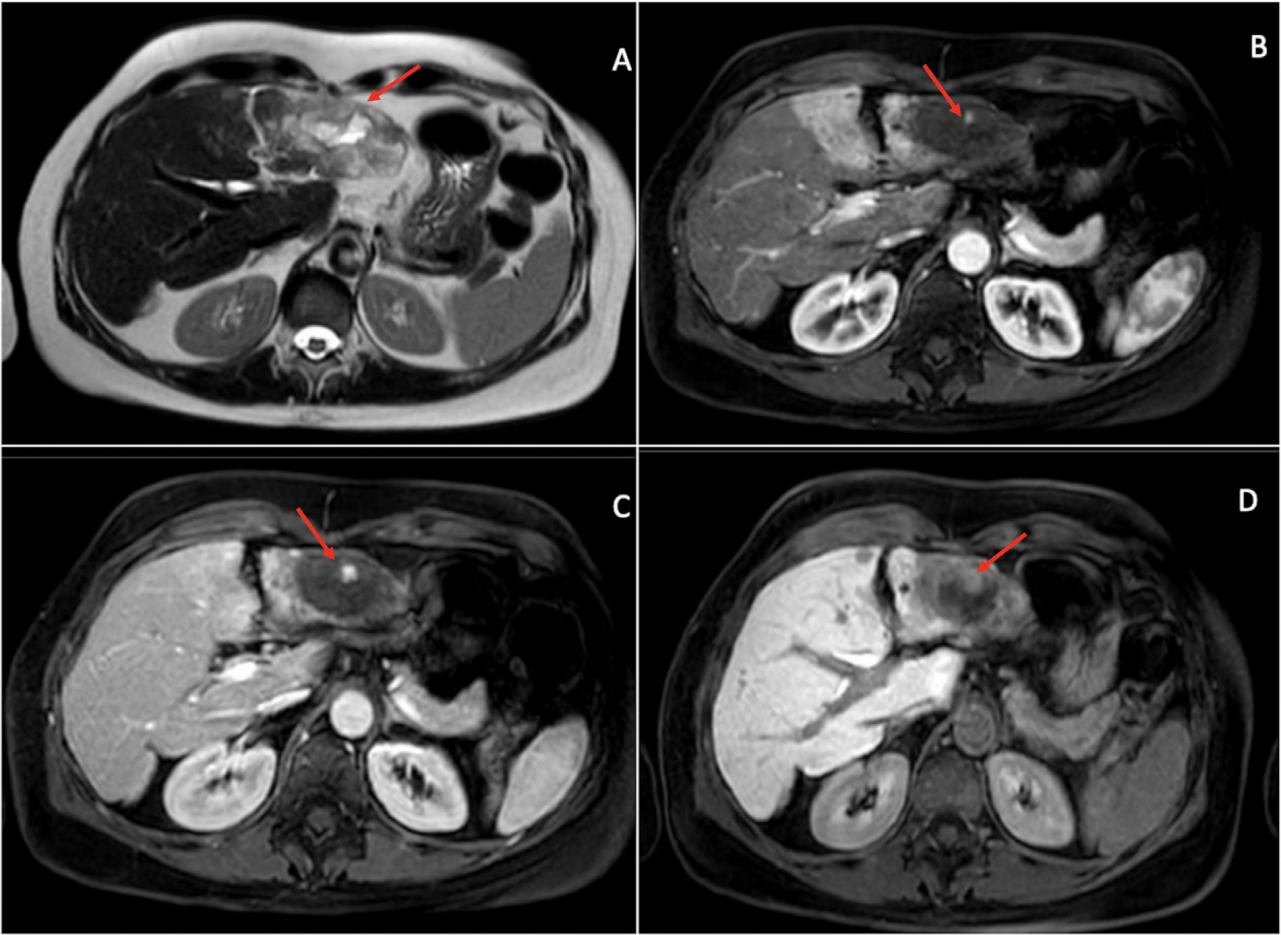
Magnetic resonance imaging with hepatospecific contrast agent. At the level of the II/III hepatic segment a non-homogeneously hyperintense signal alteration was documented in the T2-weighted sequences (A). Central vascular pooling is evident in the arterial (B) and venous (C) phases with irregular signal and centrifugal pattern. Minimal peripheral impregnation of the contrast agent was observed in the hepatobiliary phase (D).

An MRI examination was performed using a hepatobiliary-specific contrast agent (Primovist), which confirmed the presence of 2 large lesions in the II/III hepatic segment and the VII hepatic segment with a heterogeneously hyperintense signal on T2-weighted sequences (Fig. 3).

There was no evidence of areas of restricted diffusion associated with hypercellularity within the liver parenchyma.

**Fig. 4:**
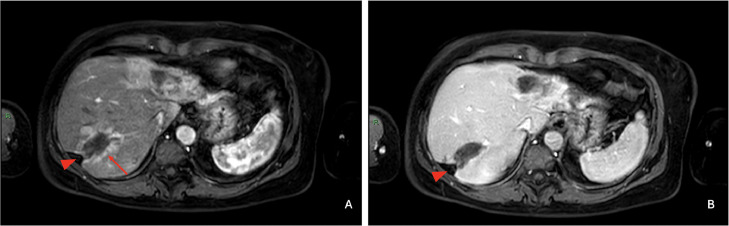
Magnetic resonance imaging with hepatospecific contrast agent. MRI showed the oblong alteration in the VII hepatic segment, causing retraction of the capsular profile (arrow head). This formation was characterized by irregular and progressive enhancement, predominantly peripheral, associated with hypervascular alteration in the arterial phase (arrow) (A) of the surrounding hepatic parenchyma, with isodensity in the later phases (B), attributed to THID (Transient Hepatic Intensity Differences).

MRI also confirmed the retraction of the capsular profile caused by the “oblong” lesion in the VII hepatic segment (Fig. 4). This lesion was characterized by irregular and progressive enhancement, predominantly peripheral. It was associated with a contiguous hypervascular alteration in the arterial phase of the surrounding hepatic parenchyma, with isointensity in the later phases, which was attributed to THID (transient hepatic intensity differences).

In the areas of peripheral hepatic parenchyma adjacent to the lesion at the II/III hepatic segment, the presence of a further THID was evident too.

Numerous centimeter-sized angiomas were observed in the remaining parenchyma.

On the hepatobiliary phase (HBP) the alteration described in the II/III hepatic segment showed minimal peripheral impregnation, appearing to be similar due to intesitometric behavior to vascular-based alteration. Conversely, the area at VII exhibited no uptake at all.

In order to exclude the presence of a malignant lesion, particularly at the VII, a further diagnostic examination was required and Positron emission tomography/computed tomography (PET-CT) with (18) F-fluorodeoxyglucose was performed.

**Fig. 5:**
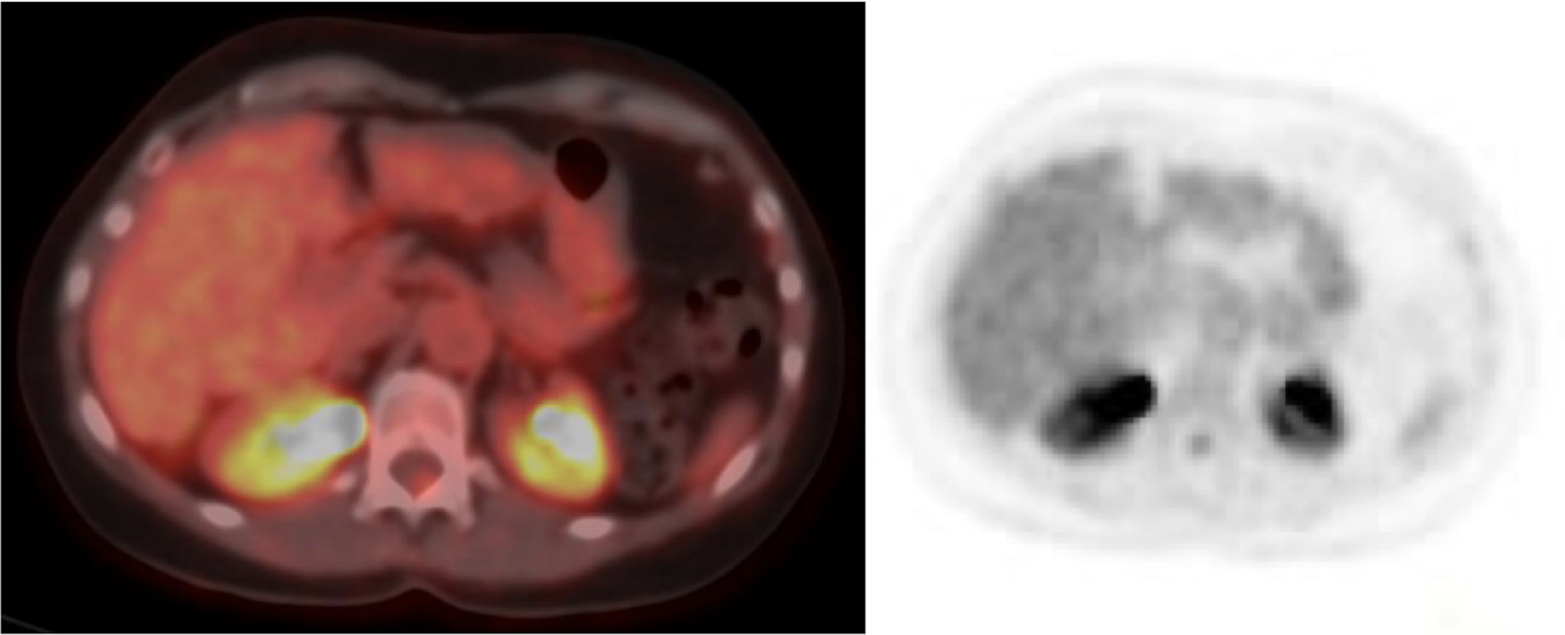
Positron emission tomography/computed tomography (PET-CT) with (18)F-fluorodeoxyglucose (18F-FDG) did not demonstrate pathological uptake of the radiopharmaceutical in the liver at the sites described in the above examination.

PET-TC with 18F-FDG scan did not reveal any pathological radiopharmaceutical uptake in the various body segments examined. Additionally, no metabolic focalities were observed in the liver at the sites described in the aforementioned examination (Fig. 5).

Although the PET-CT scan was negative, a histological examination was deemed necessary to definitively rule out malignancy and make a precise diagnosis.

An ultrasound-guided liver biopsy was then performed, targeting the nodular formation in the left lobe (segment II/III).

The histological analysis revealed liver parenchyma characterized by large cystic spaces filled with blood and sinusoidal dilatation, which was consistent with multifocal hepatic peliosis.

**Fig. 6:**
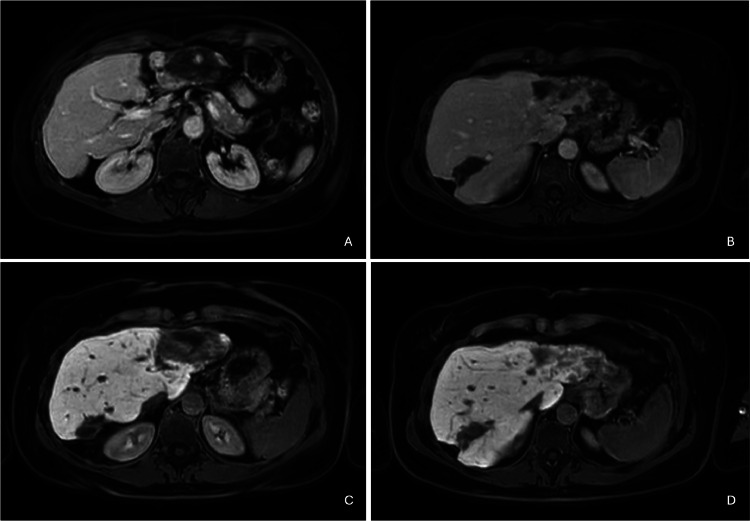
MRI performed as a three-month follow-up with hepatospecific contrast agent. Lesions at II/III and at VII hepatic segment remained unchanged on venous phase (A, B) and on hepato-biliary phase (C, D).

The patient underwent a 3-month follow-up MRI with hepato-biliary phase. The lesions described in the previous MRI remained unchanged, and as a result, no treatment was administered (Fig. 6).

## Discussion

Peliosis hepatis (PH) is a rare vascular disorder of the liver with diverse etiological agents and pathogenetic mechanisms. Histologically, PH is marked by multiple blood-filled cysts of varying sizes (<1 mm to several cm), distributed throughout the liver without a preferred location.

Two types of morphological patterns of PH are described and may coexist [7,8]: the phlebectatic variant with an endothelial lining and the parenchymal variant resulting from hepatocellular hemorrhagic necrosis.

Zak [9] proposed that the 2 morphological patterns are part of the same process. It begins with necrosis of the liver parenchyma leading to the formation of a hemorrhagic area (parenchymal pattern). This may develop into fibrous wall formation and endothelial lining (phlebectatic pattern), which may heal by fibrin deposition, thrombosis and sclerosis of the vascular spaces.

The condition is usually discovered incidentally on imaging or at autopsy, as patients are usually asymptomatic.

The pathogenesis of hepatic peliosis is not yet fully understood. However, certain drugs, particularly hormonal and antimetabolites/antineoplastic drugs, as well as medical conditions, such as malignancies, hematological disorders, transplants, diabetes, and chronic infections have been linked to its development [1,2,8].

The cause of PH in our patient was unclear. There was no history of chronic disease debilitating chronic disease (e.g., tuberculosis, hematological malignancies or AIDS), or long-term use of anabolic steroids or oral contraceptives in any of the patients. However, 20%-50% of patients with PH have no associated condition [8].

Peliosis hepatis can present as either diffuse or focal involvement of the liver. Imaging features of PH are nonspecific. CT, MRI, and PET-CT are useful diagnostic tools, but they may not always provide a definitive answer, making it difficult to distinguish PH from other liver pathologies, including malignancies [10].

CT findings of peliotic lesions can vary depending on size, presence of thrombus, and hemorrhage.

Calcifications have also been described within these lesions. On dynamic contrast-enhanced CT images, PH exhibits various enhancement patterns. During the arterial phase, peliotic lesions typically exhibit hypoenhancement, with possible globular enhancement and small collections of contrast in the center. As time progresses, the lesions become progressively isoattenuating. Some lesions may also display areas of hyperattenuation. It is possible to observe a progression of enhancement that is either centripetal or centrifugal, without a mass effect on hepatic vessels.

Distinguishing PH from hemangiomatosis, multiple abscesses, and metastases can be challenging based on CT appearance [6].

On MRI, PH lesions usually appear hyperintense on T2-weighted sequences and hypointense on T1-weighted sequences. However, their signal may vary depending on the presence and age of the blood component, and isointense and hyperintense foci have also been reported.

The enhancement pattern in MRI is similar to CT. Cystic cavities with an enhancing rim may indicate a hematoma.

Distinguishing between angiomas and PH can be difficult. PH can mimic the enhancing centripetal pattern of typical hemangiomas and atypical hemangiomas may display a centrifugal pattern.

On the HBP of gadoxetic acid-enhanced (Gd-EOB) MRI, PH has no specific pattern. Most peliotic lesions exhibit low signal intensity, with some displaying central enhancement on the hepatobiliary phase (HPB). The hypointensity signal on HBP of the peliotic lesion could confirm its vascular nature. Internal foci of signal hyperintensity may indicate the presence of normal hepatocytes spared from parenchymal destruction [5,10].

A “halo-like“ appearance has been described in certain cases of PH, appearing as a hypointense lesion with central enhancement on the hepatobiliary phase [10].

In our case, distinguishing between hepatic peliosis and other benign or malignant liver lesions, such as THID, hepatic hemangiomas or cholangiocarcinoma, was challenging.

MRI with the study of the HBP was useful to exclude differential diagnoses such as focal nodular hyperplasia (FNH), hepatocellular adenoma (HCA), and hepatic hemangiomas.

On T1-weighted MR images, FNH can appear hypointense to isointense, and on T2-weighted images, it can appear mildly hyperintense to isointense. Additionally, a hyperintense 'central scar' may be visible on T2-weighted images, which represents a collection of blood vessels, bile ducts, and fibrous tissue. FNH exhibits uniform arterial phase enhancement. The central scar appears as low signal intensity on early phase contrast-enhanced images, gradually increasing to hyperintensity relative to the rest of the lesion on delayed phase images. On HPB-MRI, FNH appears iso- or hyperintense in 94%-97% of cases [11]. In this case, the lesions did not show a preference for hepatospecific contrast medium and did not have a central scar.

Hepatocellular adenoma, like peliosis, may be associated with long-term estrogen therapy. Both can show high signal intensity on T2-weighted images, have heterogeneous enhancement and appear hypointense on HBP images. A differentiating factor is the possible presence of fat in certain adenomas.

When distinguishing between peliotic lesions and metastases or hepatocellular carcinoma (HCC) in a differential diagnosis, peliotic lesions exhibit a higher degree of hyperintensity on T2-weighted images, more prolonged contrast enhancement, and a lack of mass effect.

Additionally, malignancies like metastases and HCC typically display restricted diffusion on DWI, whereas peliosis rarely exhibits low ADC values unless there are recent internal bleeding components.

HCC may have variable signal intensity and enhancement patterns due to complications like hemorrhage, degeneration, and necrosis. The introduction of MRI studies with hepatospecific contrast agents has improved the diagnostic performance of HCC, especially in the identification and characterization of lesions with atypical features as there is a reduction in the immunohistochemical expression of the uptake transporter OATP8 during multistep hepatocarcinogenesis [12]. However, 10%-15% HCCs can show uptake of Gd-EOB on HBP, making it challenging to distinguish from other pathologies, such as peliosis [13].

Furthermore, it is essential to recognize that peliotic changes are a common occurrence in hepatocellular carcinomas (HCCs), which are referred to as ‘peliotic change’ in HCC [14].

In our case, due to the subcapsular retraction caused by the area at the level of the VII hepatic segment, it was necessary to exclude a mass-forming cholangiocarcinoma, which in CT are characterized by homogeneous attenuation, irregular peripheral enhancement with gradual centripetal enhancement and capsular retraction. Other common findings include the presence of hepatolithiasis associated with ductal dilatation and vascular invasion resulting in lobar or segmental hepatic atrophy. The MR imaging features of mass-forming cholangiocarcinoma are similar to its CT features. The mass shows an irregular margin with high signal intensity at T2-weighted imaging with low signal intensity at T1-weighted imaging. The pattern and intensity of enhancement of cholangiocarcinoma on HPB can vary and has been associated with tumor differentiation [15].

The ^18^F-FDG PET/CT can be useful to rule out malignancies. PH generally show hypometabolism or isometabolism [16].

However, it is often not possible to completely exclude malignant pathology and histology is therefore required for a definitive diagnosis. Indeed, imaging features confirmed by histological findings were the mainstay of our diagnosis.

## Conclusion

Awareness of the imaging features of hepatic peliosis is essential, as it can mimic various focal liver lesions. A multidisciplinary approach, including histological examination and imaging modalities, is crucial for accurate diagnosis and appropriate management of this rare condition.

## Compliance with ethical standards

This study was approved by the local ethics committee; moreover, it was performed in accordance with the ethical standards published in the Declaration of Helsinki of 1964 and its later amendments.

## Data sharing statement

Additional data will be available upon request from the corresponding author.

## Patient consent

Informed consent was obtained for publication of their case from the patients.
